# Variations in online self-regulated learning abilities among Chinese K-12 teachers across different regions and levels

**DOI:** 10.3389/fpsyg.2024.1463287

**Published:** 2024-10-14

**Authors:** Yan Zhao, Yu Li, Zhong Hua Sun, Qiang Jiang, Shuang Zhao

**Affiliations:** ^1^School of Education, Changchun Normal University, Changchun, China; ^2^School of Information Science and Technology, Northeast Normal University, Changchun, China; ^3^Jilin Police College, Changchun, China

**Keywords:** online self-regulated learning, K-12 teacher, educational stages, regions, education qualifications, years of service

## Abstract

**Introduction:**

Online self-regulated learning (OSRL) is crucial for online learners’ success and lifelong learning. This study investigated the OSRL characteristics of K-12 teachers in China, who embody the dual roles of learners and educators. It also analyzed the differences in OSRL abilities across different genders, education stages, and school locations, and examined the correlation between education qualifications, years of service, and OSRL abilities.

**Methods:**

A self-report measure was used to assess K-12 teachers’ OSRL, with data collected from 1,443 K-12 teachers (394 males and 1,049 females) in northeastern China. Descriptive statistical analysis was utilized to explore the characteristics of their OSRL. Independent *t*-tests and ANOVA were employed to investigate differences in OSRL among different genders, regions, and educational stages. Correlation analysis was conducted to examine the relationships between education qualifications, years of service, and OSRL among K-12 teachers.

**Results:**

The data analysis revealed that K-12 teachers scored the lowest in Online Learning Self-Efficacy (OLSE), followed by Online Learning Management Strategies (OLMS), and then Online Learning Resource Management (OLRM). Notably, urban K-12 teachers exhibited higher OLSE abilities than their rural counterparts, while high school teachers scored higher in OLSE and OLMS compared to primary school teachers. Furthermore, a positive correlation was observed between education qualifications and K-12 teachers’ OLSE, whereas a negative correlation was identified between years of service and K-12 teachers’ OLSE.

**Conclusion:**

The findings reveal an uneven development across various dimensions of online self-regulated learning among K-12 teachers, necessitating support for the advancement of OLSE, OLMS, and OLRM. Particular attention should be given to the OLSE of teachers with longer teaching years and rural teachers. Encouraging K-12 teachers with lower education qualifications to pursue further education is also recommended. This study provides evidence and a novel perspective for teacher educators to develop online professional development programs, which is significant for optimizing online learning experiences and enhancing educational outcomes.

## 1 Introduction

With the rapid advancement of information technology, online learning has gained considerable traction and has found extensive applications in the field of education (Xu and Xu, 2020). In primary and secondary schools, online learning has evolved into a pivotal tool for reshaping traditional classroom teaching. Through the integration of blended learning, it aims to optimize the efficiency and effectiveness of conventional school education, thereby enhancing students’ overall learning satisfaction. Particularly during the COVID-19 pandemic, this trend has experienced significant momentum, with numerous primary and secondary schools requiring teachers to possess the capability to conduct online teaching and seamlessly transition to online instruction as needed (Johnson et al., 2023).

Concurrently, to adapt to the digital transformation of education, K-12 teacher professional development programs need to align with the requirements of online teaching, thereby enhancing teacher competencies. These programs strive to bolster teachers’ proficiency in both online instruction and learning methodologies through the execution of online learning initiatives. Additionally, K-12 teachers can leverage fragmented time to participate in online professional development activities while engaged in online learning. This practice significantly mitigates conflicts stemming from concurrent work and academic commitments. In recent years, online learning has become a common form of professional development for K-12 teachers in China.

However, in the online learning environment, a temporal and spatial gap exists between instructors and students, resulting in a dearth of in-person communication and engagement. Online learning relies on both asynchronous and synchronous interaction within a virtual environment (Serdyukov, 2020). Compared to traditional classroom instruction, online learning necessitates a higher degree of learner autonomy (Yu, 2023). It requires learners to demonstrate elevated levels of online self-regulated learning (OSRL) abilities (Broadbent and Poon, 2015), thereby implying a heightened engagement in managing their learning processes, encompassing the establishment of learning objectives, monitoring progress, and adjusting learning strategies accordingly.

In K-12 online education, research has highlighted the importance of utilizing various technologies and methods to enhance students’ OSRL abilities (Johnson et al., 2023). However, numerous research findings indicate that students often lack robust OSRL abilities, exhibit deficiencies in pertinent OSRL knowledge, and struggle to effectively employ learning strategies (Broadbent and Poon, 2015; Kuo et al., 2020; Archambault et al., 2022). To enhance students’ OSRL abilities and optimize the efficacy of online instruction, K-12 teachers must undergo transformation, acquiring robust OSRL proficiencies and adeptness in implementing online SRL strategies (Peeters et al., 2014; Sáez-Delgado et al., 2022).

K-12 teachers play a crucial role in supporting the development of students’ self-regulating learning ability in many aspects (Dignath and Veenman, 2021; Perry et al., 2020; Sang et al., 2023). An apt Chinese proverb underscores the imperative preparedness of educators: “If you want to give the students a glass of water, the teacher should have a bucket of water.” This proverb accentuates the essentiality for teachers to possess robust OSRL abilities to effectively nurture students’ OSRL proficiencies and guide their learning pathways within educational settings (Kramarski and Heaysman, 2021). Empirical investigations corroborate that students can transition into adept online self-regulated learners under the expert guidance of K-12 teachers (Karlen et al., 2023; Heirweg et al., 2020).

Existing research indicates that while K-12 teachers, as adult learners, possess some degree of OSRL ability, their proficiency in OSRL does not meet expected levels, particularly when confronted with different online learning environments (Karlen et al., 2023). K-12 teachers exhibit disparities in two primary aspects: First, there is considerable variation in OSRL abilities among different K-12 teachers (Liu et al., 2021). Second, individual K-12 teachers demonstrate varying levels of proficiency across different components of OSRL (Alqurashi, 2016; Ogodo et al., 2021). Scholars advocate for a targeted focus on the disparities in OSRL abilities among K-12 teachers, with the aim of continuously enhancing their abilities in this area (Broadbent and Poon, 2015; Chen and Bonner, 2020).

In light of this, the OSRL abilities of K-12 teachers are indispensable. They must possess ample OSRL skills to effectively foster the growth of students’ OSRL abilities (Karlen et al., 2023; Sáez-Delgado et al., 2022; Vosniadou et al., 2020). This study aims to investigate the characteristics of OSRL abilities among K-12 teachers, analyzes the differences in OSRL abilities across different genders, educational stages, and school locations, and examines the correlation between education qualifications, years of service, and OSRL abilities.

## 2 Literature review

Self-regulated learning (SRL) refers to learners who are metacognitively, motivationally, and strategically engaged in learning (Winne, 2005). Online self-regulated learning (OSRL), conceptually, refers to the ability to autonomously manage one’s learning process by establishing objectives, tracking progress, and employing tactics aimed at improving learning outcomes within an online setting (Zimmerman, 2000; Winne, 2019). The OSRL ability is a critical factor in achieving good online learning performance for learners and is regarded as a key competency for adults to achieve lifelong learning (Broadbent and Poon, 2015).

Teachers with strong OSRL abilities are better equipped to efficiently manage online learning tasks, which in turn promotes their professional growth. Moreover, these abilities enable teachers to gain a deeper understanding of students’ OSRL experiences, allowing them to more effectively identify and address students’ learning needs and barriers, and to implement more targeted instructional interventions (Karlen et al., 2023). K-12 Teacher’s OSRL ability not only impacts their own teaching performance but also has a profound effect on students’ online learning experiences and outcomes (Xu et al., 2023).

While it is theoretically assumed that teachers, as adult learners, should possess strong OSRL skills, existing research reveals significant variability in this ability. Bylieva et al. (2021) found that teachers often struggle with time management, leading to incomplete tasks and decreased engagement. Dabbagh and Kitsantas (2013) further noted that maintaining motivation is particularly challenging for teachers in online learning environments lacking immediate feedback or peer interaction. Additionally, Greene et al. (2014) highlighted that teachers frequently experience cognitive overload when navigating new technological platforms and integrating complex content, which undermines their ability to effectively SRL abilities.

Moreover, OSRL abilities are influenced by individual characteristics, educational levels, and regional differences. Most studies suggest that females tend to perform better in OSRL, especially in traditional learning environments. Research by Wang et al. (2013) indicates that females are more adept at employing metacognitive strategies, setting learning goals, and managing time, possibly due to their stronger sense of academic responsibility and self-discipline. However, gender differences are not consistent across all cultural contexts. Aydoğmuş and Ibrahim (2022), for example, found no significant gender differences in OSRL in studies conducted in the U.S. and Turkey, highlighting the need for further investigation into these discrepancies.

The impact of individuals’ academic backgrounds and teaching experiences on their OSRL abilities also requires further exploration to uncover underlying mechanisms. Existing research suggests that teachers with higher academic qualifications generally possess stronger metacognitive skills and strategic behaviors, which enable them to more effectively self-monitor and adjust in complex online learning environments (Loeng, 2020). Additionally, extensive teaching experience allows teachers to accumulate diverse learning strategies, enhancing their ability to reflect and assess their own learning, thereby better addressing the challenges posed by online learning (Zimmerman, 2000).

Regional disparities also influence teachers’ OSRL abilities. Huh and Reigeluth (2018) found that rural teachers often have lower OSRL abilities compared to their urban counterparts, mainly due to their limited familiarity with digital tools and lack of access to technical support and training opportunities. Manner and Rodriguez (2012) further noted that rural teachers, due to fewer professional development opportunities, exhibit lower motivation and engagement in online learning, and often lack the necessary guidance and feedback, which undermines their OSRL abilities. Investigating these regional differences is essential for understanding the challenges teachers face in diverse educational environments and for improving support systems aimed at enhancing their OSRL abilities.

Therefore, research on K-12 teachers’ OSRL should employ appropriate measurement tools to assess their abilities and examine differences across regions, teacher categories, and educational levels. These insights are crucial for improving teachers’ learning outcomes and effectively advancing targeted professional development efforts.

Through a review of the literature, in measuring learners’ online self-regulated learning abilities, the analysis of self-assessment and reflection plays a major role, with questionnaires or surveys being the primary methods of data collection (Basilotta-Gómez-Pablos et al., 2022; Rovers et al., 2019). Pintrich et al. (1993) developed the Motivated Strategies for Learning Questionnaire (MSLQ), which has gained widespread usage. This instrument comprises four constructs: learning motivation (e.g., intrinsic and extrinsic goal orientation, self-efficacy), cognitive strategies (e.g., rehearsal, elaboration), metacognitive strategies (e.g., planning, monitoring), and resource management strategies (e.g., help-seeking, time management). Weinstein et al. (2002) introduced the Learning and Study Strategies Inventory (LASSI), which encompasses dimensions such as attitudes, motivation, time management, test strategies, and academic resources. Barnard et al. (2009) devised the Online Self-Regulated Learning Questionnaire, focusing on six dimensions: goal-setting, task strategies, time management, environmental structuring, help-seeking, and self-evaluation. Although each scale has its structure and items, in general aspects, some coincidences underline the importance of motivation, self-efficacy, and learning strategies.

Existing research has indicated that Online Learning Motivation (OLM) constitutes a crucial component of OSRL abilities (Deci and Ryan, 2000; Pintrich, 2003). Learners with strong motivation are better equipped to regulate their online learning processes effectively. Some researchers have initiated investigations into the influence of gender on online learning motivation (Liu et al., 2021). However, the current findings remain inconclusive, with inconsistent effects of gender on OLM observed across learners of different ages (Ajlouni et al., 2022; Yukselturk and Bulut, 2009; Yu and Deng, 2022). It is necessary to further explore whether there are differences in OLM among K-12 teachers of different genders.

Online Learning Self-Efficacy (OLSE) is defined as a learner’s confidence to complete online learning tasks and achieve favorable outcomes in online learning (Pampaka et al., 2018). Learners with high OLSE are more likely to exert the necessary effort to overcome obstacles in online learning (Mushtaque et al., 2022; Calaguas and Consunji, 2022). The OLSE is a significant predictor that can enhance academic success (Taipjutorus et al., 2012; Zimmerman and Kulikowich, 2016). Some Studies have shown that OLSE can facilitate learners’ academic performance by fostering their OSRL abilities (Dignath and Veenman, 2021; Karlen et al., 2020; De Smul et al., 2018; Usher and Pajares, 2008).

Some researchers believe the online learning experience of learners can influence their OLSE (Peechapol et al., 2018). The simplicity and user-friendliness of the online learning platform, along with learners’ digital literacy, can influence their OLSE (Yeşilyurt et al., 2016). A certain level of computer proficiency and confidence in online communication methods can influence learners’ OLSE (Xu et al., 2023). Therefore, researchers suggest that there are differences in OLSE among learners (Shen et al., 2013; Alqurashi, 2016; Wang et al., 2013). Researchers can investigate the characteristics of learners in depth to explore the nuances of their OLSE, thereby enabling targeted interventions aimed at enhancing learners’ sense of OLSE (Peechapol et al., 2018).

With the increasing prevalence of online education across higher education and K-12 settings, the adept utilization of effective SRL strategies in online environments is paramount for academic success. The necessity for online learners to exhibit traits of self-direction, independence, and autonomy has been emphasized (Schunk and Zimmerman, 2011). Researchers have increasingly explored the characteristics of learners’ online self-regulated learning from the perspectives of online learning motivation, self-efficacy, and learning strategies (Barnard et al., 2009; Pintrich, 2004). Existing research serves as an important reference for this study in exploring the OSRL abilities of K-12 teachers.

Previous research has predominantly focused on SRL abilities of students within traditional face-to-face classroom environments, often neglecting adult learners, particularly K-12 teachers. This oversight is significant, as teachers play a crucial role in fostering SRL abilities among their students. Additionally, findings related to online OSRL have not always been consistent, underscoring the pressing need for further investigation in this area. Understanding K-12 teachers’ OSRL abilities is essential for the development of effective professional development programs and the enhancement of instructional practices. The following research questions guided our investigation:

Q1.How do K-12 teachers perceive their abilities in OSRL?
Q2. Is there a difference in the perception of the OSRL abilities among K-12 teachers of different genders, educational stages (primary school, middle school, high school), and regions (urban, rural)?
Q3. Is there a correlation between the education qualifications, years of service, and the OSRL abilities of K-12 teachers?

## 3 Materials and methods

### 3.1 Procedure

The study was approved by the Science and Technology Ethics Committee of Changchun Normal University. All participants provided voluntary consent to participate. Prior to the start of the survey, participants were fully informed of the study’ objectives and explicitly informed of their right to withdraw at any time. Additionally, participants were assured that their identities would remain confidential and that all collected data would be anonymized.

Respondents utilized the Questionnaire Star software for questionnaire completion. Before responding, all participants provided informed consent. They were informed that “submitting the questionnaire” would be considered as giving consent. Overall, participants spent about 8 min to complete the electronic questionnaire.

### 3.2 Instruments

According to the literature review, researchers generally consider online self-regulated learning to encompass motivation, self-efficacy, and learning strategies. Among these, learning strategies primarily focus on cognitive learning strategies, metacognitive learning strategies, and resource management learning strategies. Therefore, this survey’s sub-dimension items were derived from published sources, translated into Chinese, and subsequently refined. The questionnaire utilized in this study comprised two sections. The first segment elicited participants’ demographic details, including gender, education qualifications, schooling stages, school location, and years of service. The second segment encompassed items gauging each sub-dimension of OSRL. Responses for all items were recorded on a 7-point Likert-type scale, ranging from 1 denoting “strongly disagree” to 7 indicating “strongly agree.”

To ensure the validity and reliability of the OSRL questionnaire, exploratory factor analysis (EFA) and confirmatory factor analysis (CFA) were conducted using SPSS 26 and AMOS 26, respectively. The EFA identified five distinct five-factor OSRL instruments, that is OLM (6 items), OLSE (7 items), OLCS (10 items), OLMS (13 items), and OLRM (7 items). The reliability (alpha) coefficients for the five factors ranged from 0.91 to 0.97. The Composite Reliability (CR) values ranged from 0.90 to 0.96. Additionally, the variables exhibited a Kaiser-Meyer-Olkin (KMO) measure of 0.966. The CFA results also supported the five-factor structure and high model fit indices (CMIN/DF = 2.57, RESEA = 0.07, TLI = 0.93, CFI = 0.94). These results signify a robust fit of the proposed model to the observed data, providing strong support for the questionnaire’s reliability and validity. The details of each scale are as follows, and further information can be found in Appendix A:

1. Online Learning Motivation (OLM) scale: evaluating K-12 teachers’ motivation in the context of online learning, encompassing both intrinsic and extrinsic motivations (Pintrich et al., 1993). In this study, the OLM scale obtained Cronbach’s alpha values of 0.91.
2. Online Learning Self-Efficacy (OLSE) scale: assessing K-12 teachers’ confidence in navigating online learning platforms and engaging in online learning interactions (Shen et al., 2013). In this study, the OLSE scale obtained Cronbach’s alpha values of 0.95.
3. Online Learning Cognitive Strategies (OLCS) scale: exploring K-12teachers’ cognitive approaches to online learning, such as paraphrasing, organization, and in-depth processing of learning material (Pintrich et al., 1993). In this study, OLCS obtained Cronbach’s alpha values of 0.96.
4. Online Learning Metacognitive Strategies (OLMS) scale: measuring K-12 teachers’ predominant use of strategies that involve the establishment of online learning objectives, self-planning throughout the learning process, self-monitoring, and metacognitive management techniques (Pintrich et al., 1993; Barnard et al., 2009). In this study, the OLMS scale obtained Cronbach’s alpha values of 0.97.
5. Online Learning Resource Management Strategies (OLRM) scale: assessing K-12 teachers’ focus on resource management tactics related to online learning time allocation, effort management, and seeking assistance when encountering learning challenges (Pintrich et al., 1993; Barnard et al., 2009). In this study, the OLRM scale obtained Cronbach’s alpha values of 0.97.

### 3.3 Participants

A hybrid approach combining stratified sampling and random sampling was adopted to recruit 500 teachers from primary, middle, and high schools in Northeast China, respectively. Incomplete responses and questionnaires with a response time of less than 3 min were excluded. Ultimately, 1,443 valid responses were collected, yielding a valid data rate of 96.2%. Among the 1,443 K12 teachers, 498 were primary school teachers, 496 were middle school teachers, and 449 were high school teachers. The demographic distribution included 72.7% female (*n* = 1,049) and 27.3% male (*n* = 394). In terms of location, 59.4% were urban teachers (*n* = 858), while 40.6% were rural teachers (*n* = 585). Regarding education qualifications, 10.1% held an associate degree (*n* = 146), 81.5% held a bachelor’s degree (*n* = 1,176), and 8.4% held a graduate degree (*n* = 121). Teaching experience was diverse, with 9.7% having 0–5 years, 11.4% having 5–10 years, 8.1% having 11–15 years, 8.1% having 16–20 years, 24.3% having 11–15 years, 21.3% having 16–20 years, 32.7% having 21–25 years, and 13.8% having more than 25 years of teaching experience.

### 3.4 Statistical analysis

The collected data followed a normal distribution, and a series of analyses were conducted using SPSS 26 software. Firstly, independent samples t-tests were conducted using SPSS 26.0 to examine the OSRL abilities of K12 teachers across different demographic categories, including gender and region. Subsequently, ANOVA was employed to investigate differences in OSRL abilities among primary, middle, and high school teachers. A significance level of *p* < 0.05 was considered statistically significant. Lastly, Pearson correlation analysis was undertaken to explore the relationships between K12 teachers’ OSRL abilities and their education qualifications as well as years of service.

## 4 Results

### 4.1 K-12 teachers’ responses on the OSRL

To address our first research question, we conducted a descriptive statistical analysis on the collected valid data about various dimensions of OSRL among K-12 teachers, as reported in Table 1. Among the five dimensions, OLSE exhibited the lowest level of personal perception (M = 4.02, SD = 0.87), followed by OLMS (M = 4.11, SD = 0.63). The mean of OLRM (M = 4.28, SD = 0.68) was slightly higher than that of OLMS. Conversely, OLM (M = 4.73, SD = 1.40) and OLCS (M = 4.68, SD = 1.04) exhibited the highest and second-highest mean levels of personal perception, respectively.

**Table 1:** Descriptive analysis of K-12 teachers’ OSRL.

Variables	*N*	Mean	SD	Range
OLSE	1,443	4.02	0.87	1.00–7.00
OLM	1,443	4.73	1.40	1.00–7.00
OLCS	1,443	4.68	1.04	1.00–7.00
OLMS	1,443	4.11	0.63	1.00–7.00
OLRM	1,443	4.28	0.68	1.00–7.00

SD, standard deviation.

### 4.2 The comparisons of K-12 teachers’ OSRL by gender, region, educational stage

To address research question 2, independent samples t-tests were employed to analyze the differences in OSRL levels among K-12 teachers of different genders (male = 1, female = 2) and regions (urban = 1, rural area = 2). Additionally, ANOVA was used to analyze differences in OSRL among K-12 teachers across various educational stages (primary school teachers = 1, middle school teachers = 2, high school teachers = 3). The findings showed no notable distinction in OSRL dimensions between male and female K-12 teachers. However, a notable finding emerged from the independent samples t-test, revealing a significant difference in OLSE between urban and rural K-12 teachers, as depicted in Table 2. The mean scores of OLSE for urban K-12 teachers (M = 4.12) were higher than those of rural K-12 teachers (M = 3.86).

**Table 2:** Comparisons of K-12 teachers’ scores of OSRL between different regions.

Variables	Region	*N*	Mean	SD	T	*p*
OLSE	Urban(1)	858	4.1235	0.86732	5.804	0.000**
	Rural(2)	585	3.8570	0.83994	5.840	0.000**
OLM	Urban(1)	858	4.7122	1.41790	−0.606	0.545
	Rural(2)	585	4.7579	1.39356	−0.608	0.544
OLCS	Urban(1)	858	4.6946	1.04728	0.657	0.512
	Rural(2)	585	4.6581	1.02271	0.660	0.510
OLMS	Urban(1)	858	4.0959	0.62613	−1.276	0.202
	Rural(2)	585	4.1392	0.64436	−1.269	0.205
OLRM	Urban(1)	858	4.2823	0.66801	0.192	0.848
	Rural(2)	585	4.2753	0.69648	0.190	0.849

*p < 0.05; **p < 0.01.

The results of the variance analysis of OSRL for K12 teachers are shown in Table 3. We found the OLSE and OLM of high school teachers were higher than that of primary school teachers (*F* = 6.50, *p* < 0.05, *F* = 3.90, *p* < 0.05), but there were no significant differences between high school teachers and middle school teachers. On the other hand, the high school teachers have higher OLSE and OLM. High school teachers are more willing to participate in online learning, and they are confident in the functionality of the platform, online communication, technology integration, and the ability to complete online learning tasks very well.

**Table 3:** Comparisons of K-12 teachers’ scores of OSRL among three educational stages.

Variables	Primary school teachers (*N* = 498) (M, S.D.)	Middle school teachers (*N* = 496) (M, S.D.)	High school teachers (*N* = 449) (M, S.D.)	F(ANOVA) Scheffe Test	
OLSE	(3.93,0.859)	(4.00,0.801)	(4.13,0.930)	6.498*	(3) > (1)
OLM	(4.62,1.308)	(4.71,1.390)	(4.87,1.520)	3.917*	(3) > (1)
OLCS	(4.64,0.991)	(4.63,1.020)	(4.67,1.037)	3.487	
OLMS	(4.12,0.629)	(4.09,0.576)	(4.13,0.698)	0.603	
OLRM	(4.32,0.663)	(4.23,0.644)	(4.30,0.732)	2.392	

*p < 0.05; **p < 0.01.

### 4.3 Correlations between K-12 teachers’ education qualifications/years of service and OSRL

To address research question 3, we conducted an analysis examining the correlation between education qualifications and years of service with OSRL among K-12 teachers. Among the respondents, 10.1% held an associate degree (*n* = 146), 81.5% held a bachelor’s degree (*n* = 1,176), and 8.4% held a graduate degree (*n* = 121). To elucidate the relationship between education qualifications and OSRL among K-12 teachers, separate Pearson correlation analyses were performed. The correlations between education qualifications and OSRL among K-12 teachers are outlined in Table 4. Results showed a positive correlation between education qualifications and OLSE (correlation coefficient = 0.129, *p* < 0.01), while no significant correlation was observed with the other four dimensions. In essence, these findings suggest that as education qualifications advance, K-12 teachers exhibit heightened self-efficacy and confidence in online learning endeavors.

**Table 4:** The correlation between the subscales of the OSRL survey and K-12 teachers’ education qualifications.

	OLSE	OLM	OLCS	OLMS	OLRM
Degree	0.129**	0.016	0.005	0.044	0.030

*p < 0.05; **p < 0.01.

The years of service among K-12 teachers were categorized into six groups: 0–5 years, 6–10 years, 11–15 years, 16–20 years, 20–25 years, and more than 25 years. To facilitate analysis, the years of service were converted into virtual continuous variables, represented by the corresponding numerical values: 1, 2, 3, 4, 5, and 6, respectively. Table 5 displays the correlations between years of service and OSRL among K-12 teachers. Results revealed a significant negative correlation between years of service and OLSE (correlation coefficient = −0.126, *p* < 0.01), with no significant correlation observed with other dimensions. This finding suggests that K-12 teachers with longer teaching experience tend to be older and may possess weaker network operation skills compared to younger teachers, which could impact their confidence in online learning to some extent.

**Table 5:** The correlation between subscales of the OSRL survey and years of service among K-12 teachers.

	OLSE	OLM	OLCS	OLMS	OLRM
Years of service	−0.126**	0.044	0.001	0.015	0.019

*p < 0.05; **p < 0.01.

## 5 Discussion

### 5.1 Low dimensions of K-12 teachers’ perception of OSRL

The results of this study indicate that the mean scores of K-12 teachers’ OSRL across dimensions range between 4 and 5 on a seven-point scale. Among the five scales of the OSRL survey, they scored relatively lower on the OLSE, OLMS, and OLRM compared to others. According to the relevant research on teachers’ OLSE, researchers proposed that teachers’ OLSE is changeable and can be developed through teacher training (Michalsky, 2021; Karlen et al., 2023). To potentially augment K-12 teachers’ OLSE, teacher educators can implement a range of strategic approaches. Initially, teacher educators should consider continuously integrating various online professional development plans tailored to K-12 educational professionals based on the characteristics and evolving trends of K-12 teachers’ OSRL. This deliberate incorporation ensures a sustained exposure of K-12 teachers to technology-enhanced learning modalities across the duration of the program (Liu et al., 2021; Basaran and Yalman, 2020). Subsequently, teacher educators can proffer targeted support mechanisms for K-12 teachers engaged in online learning endeavors. These mechanisms encompass the facilitation of online learning partnerships, the management of online learning materials, the provision of timely feedback on learning progress, and the mitigation of the apprehension and uncertainty inherent in K-12 teachers’ online learning experiences arising from the technological milieu. By perpetually bolstering K-12 teachers’ confidence in online learning and refining their self-efficacy in this domain, teacher educators can cultivate a more proficient cadre of practitioners adept at leveraging digital platforms for learning and teaching.

Besides OLSE, the OLMS and OLRM of K-12 teachers are relatively low. The OLMS refers to K-12 teachers’ self-planning, self-monitoring, and management and regulation of online goal setting, and learning process. This aligns with certain findings derived from prior research. Existing research has found that adults can monitor and reflect on their strategy usage. However, there is still a lack of metacognitive knowledge, especially in online learning environments (Anthonysamy et al., 2020). Some researchers support that the utilization of OLMS among adults is still relatively limited (Hashemyolia et al., 2015; Moreno-Marcos et al., 2019). A wealth of research indicates that OLMS is a crucial dimension of learners’ OSRL abilities, significantly impacting online learning performance (Shen and Liu, 2011; Cho and Heron, 2015; Goradia and Bugarcic, 2017; Dumford and Miller, 2018).

The OLRM primarily involves the strategic scheduling of online learning time, effective effort management, and seeking assistance when facing challenges. Observed differences in the application of OLRM strategies have been noted across traditional and online learning settings (Broadbent and Poon, 2015). The effectiveness of resource administration strategies may differ between traditional and online settings. Learners transitioning from traditional to online environments may encounter unfamiliar challenges, potentially requiring adjustments in their approach to resource management strategies. For example, when learners encounter learning difficulties in a traditional setting, they can easily seek help from teachers and peers. However, in an online learning context, interactions between teachers and learners, as well as among learners themselves, occur asynchronously in terms of time and space. Learners seeking help from teachers and peers may not receive feedback as promptly. Moreover, some learners may not know how to utilize online tools to seek assistance (Broadbent, 2017; Anthonysamy et al., 2020).

Therefore, K-12 teachers can be supported through targeted interventions in online professional development programs to promote the development of OLMS and OLRM. Educational administration departments should establish avenues for K-12 teachers to enhance their knowledge, skills, and practical experience, enabling them to effectively acquire and refine OLMS (Anthonysamy et al., 2021; Kasalak and Dağyar, 2020). Additionally, we recommend that online learning platforms integrate intelligent feedback systems and real-time assessment tools, while visually presenting K-12 teachers’ learning paths and role models to encourage reflection on their online learning and timely adjustments to their learning pace. Support teachers can provide scaffolding for K-12 teachers engaged in online learning, such as learning plan prompts, reflection frameworks, and personalized learning path recommendations, to continuously enhance their OLMS. Moreover, teacher educators should focus on improving K-12 teachers’ OLRM by utilizing intelligent agents, recommending learning partners, offering time management tools, and providing regular learning reminder emails to strengthen their OLRM abilities.

### 5.2 Comparison of K-12 teachers’ OSRL by gender, region, stage

The findings of this study indicate that gender does not significantly influence the OSRL abilities of K-12 teachers. The previous research findings regarding whether gender affects learners’ SRL have been inconsistent. Some studies have found no gender disparities in SRL among US or Turkish samples (Aydoğmuş and Ibrahim, 2022; Liu, 2017). Existing literature examination reveals a consistent trend where past studies on students frequently indicate that females exhibit higher levels of self-regulated learning abilities than males, especially in traditional learning environments (Wang et al., 2022; Zheng et al., 2022). Furthermore, K-12 teachers have been exposed to pedagogical training and instructional methodology throughout their professional development, a distinction that sets them apart from students (De Smul et al., 2018). The variability in research conclusions can primarily be ascribed to differences in study populations and their respective educational environments, encompassing both online and traditional learning settings (Cheng et al., 2023).

The results of the differential analysis suggest that there are disparities in OLSE between urban and rural K-12 teachers, with urban teachers demonstrating higher levels compared to their rural counterparts. In alignment with the viewpoints of existing research, some studies suggest that due to remote geographical locations and inadequate hardware facilities, rural K-12 teachers exhibit relatively lower levels of OLSE. This is reflected in their lack of confidence in utilizing technology for both learning and teaching purposes (Kellerer et al., 2014; Ogodo et al., 2021). Some researchers believe the online learning experience of learners can influence their OLSE (Moos and Azevedo, 2009; Peechapol et al., 2018) Meanwhile, the simplicity and user-friendliness of the online learning platform, along with learners’ digital literacy, can influence their OLSE (Prior et al., 2016; Tsai et al., 2011; Yeşilyurt et al., 2016). Additionally, students’ digital literacy also impacts K-12 teachers’ OLSE in rural areas, where online interactions rely on digital tools. Teachers must not only possess strong digital skills but also address disparities in student proficiency (Xu et al., 2023). In rural regions, limited access to devices and technology often leads to lower student digital literacy, which may undermine teachers’ confidence in delivering effective online instruction (Kellerer et al., 2014).

Therefore, the measures are required to enhance K-12 teachers’ OLSE, with a particular emphasis on rural K-12 teachers. This involves improving hardware facilities in rural schools and implementing online learning support strategies to aid rural K-12 teachers in boosting their OLSE.

Data analysis indicates that high school teachers demonstrate significantly higher levels of OLSE and OLM compared to their counterparts in primary education. Self-reports from high school teachers suggest a prevalent perception that their professional responsibilities are demanding, often necessitating extended hours on campus each day. They perceive online learning as offering flexible scheduling, which minimally disrupts their routine teaching duties. Furthermore, in high schools, there is a relatively higher proportion of young teachers with advanced education qualifications, combined with their proficient use of information technology, which notably contributes to the cultivation of their self-efficacy and motivation towards online learning.

On the other hand, in primary school settings, the developmental stage of students may require the adoption of hands-on and personalized teaching methods. Overcoming challenges associated with young students, coupled with limitations on the use of computer devices in the classroom to protect children’s visual health, typically restricting teachers’ use of technology to no more than 20 min. Consequently, these limitations on accessing and applying online resources may impact the self-efficacy and motivation of primary school teachers in online learning.

### 5.3 K-12 teachers’ OSRL in relation to education qualifications and years of service

The correlation results of this study indicate that the teachers’ education qualifications are positively correlated with OLSE. That is, K-12 teachers with higher qualifications tended to perceive themselves as having higher levels of OLSE. In this study, survey data demonstrates that among the 121 teachers with graduate degrees, 109 are high school teachers, accounting for as much as 90%. This reciprocal validation reinforces the conclusion drawn from our study, suggesting that high school teachers demonstrate a high level of OLSE. Moreover, this conclusion is also consistent with previous research. Prior studies have shown that K-12 teachers with advanced education qualifications tend to exhibit higher levels of OLSE and are perceived as more proficient in effectively integrating technology into teaching and learning practices (Liang et al., 2013; Lee et al., 2020; Peechapol et al., 2018).

Results showed a negative association between K-12 teachers’ years of service and OLSE, suggesting that teachers with more teaching experience in K-12 settings tend to exhibit lower levels of personal confidence in OSRL. This finding aligns with prior research, which has similarly observed a negative relationship between technology-related self-efficacy and either years of service or age (Lin et al., 2018; Cardullo et al., 2021). Concurrently, K-12 teachers with longer years of teaching experience typically correspond to older individuals. These teachers have amassed substantial teaching experience and refined pedagogical techniques. However, their receptivity to emerging technologies diminishes, and they encounter greater challenges in adapting to new tools, environments, and online resources (Pham et al., 2023).

In future online teacher professional development programs, we anticipate a focused emphasis on the K-12 teachers with long years of teaching experience, particularly those situated in rural areas, to furnish them with timely online learning support and assistance. This proactive approach aims to mitigate the challenge of inadequate online self-regulated learning abilities stemming from diminished online learning efficacy.

## 6 Conclusion

This study validated a survey aimed at assessing K-12 teachers’ OSRL abilities. The obtained five-factor structure indicates the survey’s validity and reliability in profiling K-12 teachers’ OSRL. Additionally, survey findings revealed that K-12 teachers’ perceptions of OLSE score the lowest, followed by OLMS, and then OLRM. OLSE emerges as a critical factor influencing K-12 teachers’ OSRL abilities. Urban K-12 teachers demonstrate higher OLSE abilities compared to rural counterparts, while high school teachers exhibit higher OLSE and OLM scores compared to primary school teachers. Moreover, there is a positive correlation between education qualifications and K-12 teachers’ OLSE, while there is a negative correlation between years of service and K-12 teachers’ OLSE. This study further discusses the aforementioned findings and offers insights and recommendations. Our research provides evidence and novel perspectives for conducting targeted online professional development programs tailored to K-12 teachers’ needs.

## 7 Limitations and future research

It is acknowledged that this study has limitations. First, the samples from Northeast China were primarily selected to explore K-12 teachers’ OSRL abilities. While this region possesses rich cultural and social backgrounds that offer valuable insights for research, relying solely on samples from this region does pose certain limitations. There are significant differences in economic development, cultural customs, social structure, and other aspects between Northeast China and other regions, which may limit the applicability of research conclusions in other regions. In the future, the geographical scope of the samples should be broadened. During sample selection, the geographic distribution of samples should be expanded to cover as many different regions in China as possible. Furthermore, when conditions permit, the number of samples should be increased as much as possible to strengthen the statistical power of the study. This can be achieved by broadening the survey scope, extending the survey duration, or adopting more efficient data collection methods.

Second, it is a potential limitation that mainly self-reporting instruments were used to assess K-12 teachers’ OSRL. While surveys are an effective means of data collection, they may not fully capture the complexity and multifaceted nature of the research question. Future studies should consider incorporating a variety of research methods. For instance, cross-sectional research can capture sample characteristics at different points in time, offering a broader understanding of the factors influencing the development of online self-regulated learning abilities among K-12 teachers and generating more comprehensive data.

This study has discovered that OSRL abilities are crucial for the professional development of K-12 teachers and contribute to their ability to cultivate students’ OSRL skills in teaching practice. Investigating the OSRL abilities of K-12 teachers not only expands the research scope of self-regulated learning but also provides essential evidence for enhancing the OSRL proficiency of K-12 teachers. In turn, this can assist teacher educators in designing effective online teaching support to enable K-12 teachers with diverse backgrounds to develop their OSRL abilities. In-depth research in this area will help fill existing gaps in academic literature, promote the professional development of teachers within online teaching environments, and offer stronger support for students’ academic achievements.

## Data Availability

The raw data supporting the conclusions of this article will be made available by the authors, without undue reservation.

## References

[ref1] Ajlouni A. Rawadieh S. AlMahaireh A. Awwad F. A. (2022). Gender differences in the motivational profile of undergraduate students in light of self-determination theory: the case of online learning setting. J. Soc. Stud. Educ. Res. 13, 75–103. Available at: https://www.learntechlib.org/p/222875/

[ref2] Alqurashi E. (2016). Self-efficacy in online learning environments: a literature review. Contemp. Issues Educ. Res. (Online) 9, 45–52. doi: 10.19030/cier.v9i1.9549

[ref3] Anthonysamy L. Ah Choo K. Soon Hin H. (2021). Investigating self-regulated learning strategies for digital learning relevancy. Malays. J. Learn. Instr. 18, 29–64. doi: 10.32890/mjli2021.18.1.2

[ref4] Anthonysamy L. Koo A.-C. Hew S.-H. (2020). Self-regulated learning strategies and non-academic outcomes in higher education blended learning environments: a one-decade review. Educ. Inf. Technol. 25, 3677–3704. doi: 10.1007/s10639-020-10134-2

[ref5] Archambault L. Leary H. Rice K. (2022). Pillars of online pedagogy: a framework for teaching in online learning environments. Educ. Psychol. 57, 178–191. doi: 10.1080/00461520.2022.2051513

[ref6] Aydoğmuş M. Ibrahim M. (2022). Two approaches to investigate preservice teachers’ TPACK competencies and self-regulated learning skills in Turkiye and the United States. J. Comput. Educ. Res. 10, 531–546. doi: 10.18009/jcer.1107419

[ref7] Barnard L. Lan W. Y. To Y. M. Paton V. O. Lai S. L. (2009). Measuring self-regulation in online and blended learning environments. Internet High. Educ. 12, 1–6. doi: 10.1016/j.iheduc.2008.10.005

[ref8] Basaran B. Yalman M. (2020). Examining preservice teachers' levels of self-efficacy perceptions regarding web. Int. J. Inf. Learn. Technol. 37, 153–178. doi: 10.1108/IJILT-11-2019-0105

[ref9] Basilotta-Gómez-Pablos V. Matarranz M. Casado-Aranda L. A. Otto A. (2022). Teachers’ digital competencies in higher education: a systematic literature review. Int. J. Educ. Technol. High. Educ. 19:8. doi: 10.1186/s41239-021-00312-8

[ref10] Broadbent J. (2017). Comparing online and blended learner’s self-regulated learning strategies and academic performance. Internet High. Educ. 33, 24–32. doi: 10.1016/j.iheduc.2017.01.004

[ref11] Broadbent J. Poon W. L. (2015). Self-regulated learning strategies & academic achievement in online higher education learning environments: a systematic review. Internet High. Educ. 27, 1–13. doi: 10.1016/j.iheduc.2015.04.007

[ref12] Bylieva D. Hong J. C. Lobatyuk V. Nam T. (2021). Self-regulation in e-learning environment. Educ. Sci. 11:785. doi: 10.3390/educsci11120785

[ref13] Calaguas N. P. Consunji P. M. P. (2022). A structural equation model predicting adults’ online learning self-efficacy. Educ. Inf. Technol. 27, 6233–6249. doi: 10.1007/s10639-021-10871-y, PMID: 35002467 PMC8727476

[ref14] Cardullo V. Wang C.-H. Burton M. Dong J. (2021). K-12 teachers’ remote teaching self-efficacy during the pandemic. J. Res. Innov. Teach. Learn. 14, 32–45. doi: 10.1108/JRIT-10-2020-0055

[ref15] Chen P. P. Bonner S. M. (2020). A framework for classroom assessment, learning, and self-regulation. Assess. Educ. Principles Policy Pract. 27, 373–393. doi: 10.1080/0969594X.2019.1619515

[ref16] Cheng Z. Wang H. Zhu X. West R. E. Zhang Z. Xu Q. (2023). Open badges support goal setting and self-efficacy but not self-regulation in a hybrid learning environment. Comput. Educ. 197:104744. doi: 10.1016/j.compedu.2023.104744

[ref17] Cho M. H. Heron M. L. (2015). Self-regulated learning: the role of motivation, emotion, and use of learning strategies in students’ learning experiences in a self-paced online mathematics course. Distance Educ. 36, 80–99. doi: 10.1080/01587919.2015.1019963

[ref18] Dabbagh N. Kitsantas A. (2013). The role of social media in self-regulated learning. Int. J. Web Based Communities 9, 256–273. doi: 10.1504/IJWBC.2013.053248

[ref19] De Smul M. Heirweg S. Van Keer H. Devos G. Vandevelde S. (2018). How competent do teachers feel instructing self-regulated learning strategies? Development and validation of the teacher self-efficacy scale to implement self-regulated learning. Teach. Teach. Educ. 71, 214–225. doi: 10.1016/j.tate.2018.01.001

[ref20] Deci E. L. Ryan R. M. (2000). The “what” and “why” of goal pursuits: human needs and the self-determination of behavior. Psychol. Inq. 11, 227–268. doi: 10.1207/S15327965PLI1104_01

[ref21] Dignath C. Veenman M. V. (2021). The role of direct strategy instruction and indirect activation of self-regulated learning—evidence from classroom observation studies. Educ. Psychol. Rev. 33, 489–533. doi: 10.1007/s10648-020-09534-0

[ref22] Dumford A. D. Miller A. L. (2018). Online learning in higher education: exploring advantages and disadvantages for engagement. J. Comput. High. Educ. 30, 452–465. doi: 10.1007/s12528-018-9179-z

[ref23] Goradia T. Bugarcic A. (2017). A social cognitive view of self-regulated learning within online environment. Adv. Integr. Med. 4, 5–6. doi: 10.1016/j.aimed.2017.05.001

[ref24] Greene J. A. Seung B. Y. Copeland D. Z. (2014). Measuring critical components of digital literacy and their relationships with learning. Comput. Educ. 76, 55–69. doi: 10.1016/j.compedu.2014.03.008

[ref25] Hashemyolia S. Asmuni A. Ayub A. F. M. Daud S. M. Shah J. A. (2015). Motivation to use self-regulated learning strategies in learning management system amongst science and social science undergraduates. Asian Soc. Sci. 11:49. doi: 10.5539/ass.v11n3p49

[ref26] Heirweg S. De Smul M. Merchie E. Devos G. Van Keer H. (2020). Mine the process: investigating the cyclical nature of upper primary school students’ self-regulated learning. Instr. Sci. 48, 337–369. doi: 10.1007/s11251-020-09519-0

[ref27] Huh Y. Reigeluth C. M. (2018). Online K-12 teachers’ perceptions and practices of supporting self-regulated learning. J. Educ. Comput. Res. 55, 1129–1153. doi: 10.1177/0735633117699231

[ref28] Johnson C. C. Walton J. B. Strickler L. Elliott J. B. (2023). Online teaching in K-12 education in the United States: a systematic review. Rev. Educ. Res. 93, 353–411. doi: 10.3102/00346543221105550

[ref29] Karlen Y. Hertel S. Hirt C. N. (2020). Teachers’ professional competencies in self-regulated learning: an approach to integrate teachers’ competencies as self-regulated learners and as agents of self-regulated learning in a holistic manner. Front. Educ. 5:159. doi: 10.3389/feduc.2020.00159

[ref30] Karlen Y. Hirt C. N. Jud J. Rosenthal A. Eberli T. D. (2023). Teachers as learners and agents of self-regulated learning: the importance of different teachers competence aspects for promoting metacognition. Teach. Teach. Educ. 125:104055. doi: 10.1016/j.tate.2023.104055

[ref31] Kasalak G. Dağyar M. (2020). University student satisfaction, resource management and metacognitive learning strategies. Teach. Curriculum 20, 73–85. doi: 10.15663/tandc.v20i1.34

[ref32] Kellerer P. Kellerer E. Werth E. Werth L. Montgomery D. Clyde R.. (2014). Transforming K-12 rural education through blended learning: Teacher perspectives. Vienna, VA: International Association for K-12 Online Learning. Available at: http://www.virtuallearningalliance.org/wp-content/uploads/2017/01/Transforming-K12-Rural-Education-through-Blended-Learning-Teacher-Perspectives-December2014-1.pdf

[ref33] Kramarski B. Heaysman O. (2021). A conceptual framework and a professional development model for supporting teachers’ “triple SRL–SRT processes” and promoting students’ academic outcomes. Educ. Psychol. 56, 298–311. doi: 10.1080/00461520.2021.1985502

[ref34] Kuo Y. C. Tseng H. Kuo Y. T. (2020). “Internet self-efficacy, self-regulation, and student performance: African-American adult students in online learning” in Proceedings of International Journal on E-Learning 2020. ed. Marks G. (Waynesville, NC USA: Association for the Advancement of Computing in Education (AACE)), 161–180.

[ref35] Lee D. Watson S. L. Watson W. R. (2020). The relationships between self-efficacy, task value, and self-regulated learning strategies in massive open online courses. Int. Rev. Res. Open Distrib. Learn. 21, 23–39. doi: 10.19173/irrodl.v20i5.4389

[ref36] Liang J. C. Chai C. S. Koh J. H. L. Yang C. J. Tsai C. C. (2013). Surveying in-service preschool teachers' technological pedagogical content knowledge. Australas. J. Educ. Technol. 29, 581–594. doi: 10.14742/ajet.299

[ref37] Lin X.-F. Liang J.-C. Tsai C.-C. Hu Q. (2018). The moderating role of self-regulated learning in job characteristics and attitudes towards web-based continuing learning in the airlines workplace. Australas. J. Educ. Technol. 34, 102–115. doi: 10.14742/ajet.3198

[ref38] Liu S. H. (2017). Relationship between the factors influencing online help-seeking and self-regulated learning among Taiwanese preservice teachers. Comput. Hum. Behav. 72, 38–45. doi: 10.1016/j.chb.2017.02.034

[ref39] Liu X. He W. Zhao L. Hong J. C. (2021). Gender differences in self-regulated online learning during the COVID-19 lockdown. Front. Psychol. 12:752131. doi: 10.3389/fpsyg.2021.752131, PMID: 34603169 PMC8481384

[ref40] Loeng S. (2020). Self-directed learning: a core concept in adult education. Educ. Res. Int. 2020, 1–12. doi: 10.1155/2020/3816132

[ref41] Manner J. C. Rodriguez D. (2012). Rural redesign: Delivering online professional development for rural teachers of ESL. Online Submission.

[ref42] Michalsky T. (2021). Preservice and inservice teachers' noticing of explicit instruction for self-regulated learning strategies. Front. Psychol. 12, 1–12. doi: 10.3389/fpsyg.2021.630197, PMID: 33841259 PMC8032886

[ref43] Moos D. C. Azevedo R. (2009). Learning with computer-based learning environments: a literature review of computer self-efficacy. Rev. Educ. Res. 79, 576–600. doi: 10.3102/0034654308326083

[ref44] Moreno-Marcos P. M. Muñoz-Merino P. J. Maldonado-Mahauad J. Pérez-Sanagustín M. Alario-Hoyos C. Delgado Kloos C. (2019). Temporal analysis for dropout prediction using self-regulated learning strategies in self-paced MOOCs. Comput. Educ. 145:103728. doi: 10.1016/j.compedu.2019.103728

[ref45] Mushtaque I. Waqas H. Awais-E-Yazdan M. (2022). The effect of technostress on the teachers’ willingness to use online teaching modes and the moderating role of job insecurity during COVID-19 pandemic in Pakistan. Int. J. Educ. Manag. 36, 63–80. doi: 10.1108/IJEM-07-2021-0291

[ref46] Ogodo J. A. Simon M. Morris D. Akubo M. (2021). Examining K-12 teachers’ digital competency and technology self-efficacy during COVID-19 pandemic. J. High. Educ. Theory Pract. 21, 13–21. doi: 10.33423/jhetp.v21i11.4660

[ref47] Pampaka M. Swain D. Jones S. Williams J. Edwards M. Wo L. (2018). Validating constructs of learners’ academic self-efficacy for measuring learning gain. High. Educ. Pedagog. 3, 118–144. doi: 10.1080/23752696.2018.1454264

[ref48] Peechapol C. Na-Songkhla J. Sujiva S. Luangsodsai A. (2018). An exploration of factors influencing self-efficacy in online learning: a systematic review. Int. J. Emerg. Technol. Learn. 13, 64–86. doi: 10.3991/ijet.v13i09.8351

[ref49] Peeters J. De Backer F. Reina V. R. Kindekens A. Buffel T. Lombaerts K. (2014). The role of teachers’ self-regulatory capacities in the implementation of self-regulated learning practices. Procedia. Soc. Behav. Sci. 116, 1963–1970. doi: 10.1016/j.sbspro.2014.01.504

[ref50] Perry N. E. Lisaingo S. Yee N. Parent N. Wan X. Muis K. (2020). Collaborating with teachers to design and implement assessments for self-regulated learning in the context of authentic classroom writing tasks. Assess. Educ.: Princ. Policy Pract. 27, 416–443. doi: 10.1080/0969594X.2020.1801576

[ref51] Pham K. T. Thi Do L. H. Dinh H. V. T. Nguyen Q. A. T. Phan Q. N. Ha X. V. (2023). Professional development of primary school teachers in Vietnamese educational reform context: an analysis from a sociocultural perspective. Education 3–13 52, 428–443. doi: 10.1080/03004279.2023.2168502

[ref52] Pintrich P. R. (2003). A motivational science perspective on the role of student motivation in learning and teaching contexts. J. Educ. Psychol. 95, 667–686. doi: 10.1037/0022-0663.95.4.667

[ref53] Pintrich P. R. (2004). A conceptual framework for assessing motivation and self-regulated learning in college students. Educ. Psychol. Rev. 16, 385–407. doi: 10.1007/s10648-004-0006-x

[ref54] Pintrich P. R. Smith D. A. Garcia T. McKeachie W. J. (1993). Reliability and predictive validity of the motivated strategies for learning questionnaire (MSLQ). Educ. Psychol. Meas. 53, 801–813. doi: 10.1177/0013164493053003024

[ref55] Prior D. D. Mazanov J. Meacheam D. Heaslip G. Hanson J. (2016). Attitude, digital literacy and self efficacy: flow-on effects for online learning behavior. Internet High. Educ. 29, 91–97. doi: 10.1016/j.iheduc.2016.01.001

[ref56] Rovers S. F. Clarebout G. Savelberg H. H. de Bruin A. B. van Merriënboer J. J. G. (2019). Granularity matters: comparing different ways of measuring selfregulated learning. Metacogn. Learn. 14, 1–19. doi: 10.1007/s11409-019-09188-6

[ref57] Sáez-Delgado F. López-Angulo Y. Mella-Norambuena J. Baeza-Sepúlveda C. Contreras-Saavedra C. Lozano-Peña G. (2022). Teacher self-regulation and its relationship with student self-regulation in secondary education. Sustain. For. 14, 1–21. doi: 10.3390/su142416863

[ref58] Sang G. Wang K. Li S. Xi J. Yang D. (2023). Effort expectancy mediate the relationship between instructors’ digital competence and their work engagement: evidence from universities in China. Educ. Technol. Res. Dev. 71, 99–115. doi: 10.1007/s11423-023-10205-4, PMID: 36785812 PMC9907197

[ref59] Schunk D. H. Zimmerman B. (Eds.) (2011). Handbook of self-regulation of learning and performance. London: Taylor & Francis.

[ref60] Serdyukov P. (2020). “Asynchronous/synchronous learning chasm” in Exploring online learning through synchronous and asynchronous instructional methods. ed. Serdyukov P. (Hershey, Pennsylvania: IGI Global), 1–33.

[ref61] Shen D. Cho M. H. Tsai C. L. Marra R. (2013). Unpacking online learning experiences: online learning self-efficacy and learning satisfaction. Internet High. Educ. 19, 10–17. doi: 10.1016/j.iheduc.2013.04.001

[ref62] Shen C. Y. Liu H. C. (2011). Metacognitive skills development: a web-based approach in higher education. Turk. Online J. Educ. Technol. 10, 140–150.

[ref63] Taipjutorus W. Hansen S. Brown M. (2012). Investigating a relationship between learner control and self-efficacy in an online learning environment. J. Open Flex. Distance Learn. 16, 56–69. doi: 10.61468/jofdl.v16i1.95

[ref64] Tsai C. C. Chuang S. C. Liang J. C. Tsai M. J. (2011). Self-efficacy in internet-based learning environments: a literature review. J. Educ. Technol. Soc. 14, 222–240. Available at: http://www.jstor.org/stable/jeductechsoci.14.4.222

[ref65] Usher E. L. Pajares F. (2008). Self-efficacy for self-regulated learning: a validation study. Educ. Psychol. Meas. 68, 443–463. doi: 10.1177/0013164407308475

[ref66] Vosniadou S. Lawson M. J. Wyra M. Van Deur P. Jeffries D. Darmawan I. (2020). Pre-service teachers' beliefs about learning and teaching and about the self-regulation of learning: a conceptual change perspective. Int. J. Educ. Res. 99:101495. doi: 10.1016/j.ijer.2019.101495

[ref67] Wang Y. Cao Y. Gong S. Wang Z. Li N. Ai L. (2022). Interaction and learning engagement in online learning: the mediating roles of online learning self-efficacy and academic emotions. Learn. Individ. Differ. 94:102128. doi: 10.1016/j.lindif.2022.102128

[ref68] Wang C. H. Shannon D. M. Ross M. E. (2013). Students’ characteristics, self-regulated learning, technology self-efficacy, and course outcomes in online learning. Distance Educ. 34, 302–323. doi: 10.1080/01587919.2013.835779

[ref69] Weinstein C. E. Palmer D. R. Schultz A. C. (2002). Lassi. User’s manual for those administering learning and study strategies inventory.

[ref70] Winne P. H. (2005). A perspective on state-of-the-art research on self-regulated learning. Instr. Sci. 33, 559–565. doi: 10.1007/s11251-005-1280-9

[ref71] Winne P. H. (2019). Paradigmatic dimensions of instrumentation and analytic methods in research on self-regulated learning. Comput. Hum. Behav. 96, 285–289. doi: 10.1016/j.chb.2019.03.026

[ref72] Xu D. Xu Y. (2020). “The ambivalence about distance learning in higher education: challenges, opportunities, and policy implications” in Higher education: handbook of theory and research, vol. 35. ed. Perna L. W. (Switzerland AG: Springer Nature), 351–401.

[ref73] Xu Z. Zhao Y. Liew J. Zhou X. Kogut A. (2023). Synthesizing research evidence on self-regulated learning and academic achievement in online and blended learning environments: a scoping review. Educ. Res. Rev. 39:100510. doi: 10.1016/j.edurev.2023.100510

[ref74] Yeşilyurt E. Ulaş A. H. Akan D. (2016). Teacher self-efficacy, academic self-efficacy, and computer self-efficacy as predictors of attitude toward applying computer-supported education. Comput. Hum. Behav. 64, 591–601. doi: 10.1016/j.chb.2016.07.038

[ref75] Yu B. (2023). Self-regulated learning: a key factor in the effectiveness of online learning for second language learners. Front. Psychol. 13:1051349. doi: 10.3389/fpsyg.2022.1051349, PMID: 36710757 PMC9877336

[ref76] Yu Z. Deng X. (2022). A meta-analysis of gender differences in e-learners' self-efficacy, satisfaction, motivation, attitude, and performance across the world. Front. Psychol. 13:897327. doi: 10.3389/fpsyg.2022.897327, PMID: 35664150 PMC9159470

[ref77] Yukselturk E. Bulut S. (2009). Gender differences in self-regulated online learning environment. J. Educ. Technol. Soc. 12, 12–22.

[ref78] Zheng X. Luo L. Liu C. (2022). Facilitating undergraduates’ online self-regulated learning: the role of teacher feedback. Asia Pac. Educ. Res. 32, 805–816. doi: 10.1007/s40299-022-00697-8

[ref79] Zimmerman B. J. (2000). “Attaining self-regulation: a social cognitive perspective” in Handbook of self-regulation. eds. Boekaerts M. Pintrich P. R. Zeidner M. (San Diego, California: Academic Press), 13–39.

[ref80] Zimmerman W. A. Kulikowich J. M. (2016). Online learning self-efficacy in students with and without online learning experience. Am. J. Dist. Educ. 30, 180–191. doi: 10.1080/08923647.2016.1193801

