# Supplementary material for: Variations in online self-regulated learning abilities among Chinese K-12 teachers across different regions and levels
Source: Front Psychol. 2024 Oct 14;15:1463287. doi: 10.3389/fpsyg.2024.1463287 (PMC11519984; doi:10.3389/fpsyg.2024.1463287)
Supplement: Supplementary file 1 [file Table_1.DOCX]

**Appendix A**

**Online Learning Motivation (OLM) ：**

Q1 Compared to traditional teacher training, I prefer online forms of professional development.

Q2 Participation in online learning can enrich my theoretical and practical knowledge of teaching.

Q3 Participation in online learning can help resolve the conflict between my work and study.

Q4 Participation in online learning makes me more competitive in job title evaluations and promotions.

Q5 I feel happy when my learning achievements can be showcased during the online learning process.

Q6 In participating in online professional development, I am eager to achieve good grades.

**Online Learning Self-Efficacy (OLSE) ：**

Q1 During online learning, I am confident in successfully completing my learning tasks on the online learning platform.

Q2 During online learning, I am confident in performing operations related to the online learning platform.

Q3 When engaging in online learning, I am confident in quickly understanding the functions of the learning platform.

Q4 In an online learning environment, I am confident in uploading and downloading files.

Q5 In an online learning environment, I am confident in using online communication tools (such as email, forums, etc.) to communicate with others.

Q6 During online learning, I am confident in expressing my thoughts by posting discussion threads.

Q7 In an online learning environment, I am confident in asking questions in the discussion areas of the online learning platform and interacting with fellow teachers regarding these questions.

**Online Learning Cognitive Strategies (OLCS) ：**

Q1 Before starting online learning, I repeatedly read the learning guidance materials.

Q2 During online learning, I use visualization tools (such as tables, diagrams, concept maps, and mind maps) to help myself understand the content.

Q3 During online learning, I am willing to use the online note-taking features provided by the learning platform.

Q4 During online learning, I repeatedly watch the useful knowledge content to deepen my understanding.

Q5 Before taking periodic quizzes during online learning, I watch expert video explanations to reinforce my knowledge.

Q6 During online learning, when learning new teaching theories, I try to relate and compare them with existing theories.

Q7 Even if the tutor does not require it, I choose to study the supplementary resources provided on the online learning platform.

Q8 When learning new knowledge, I often replay video lectures to review previously learned content.

Q9 I try to apply the educational theories learned during online learning to my teaching practice to optimize teaching outcomes.

Q10 When learning important educational theories during online learning, I usually prefer to express them in my own words to deepen my understanding.

**Online Learning Metacognitive Strategies (OLMS)：**

Q1 During online learning, I develop specific study plans according to the characteristics of the learning tasks.

Q2 During online learning, I can monitor my learning progress.

Q3 During online learning, I can consciously urge myself to study.

Q4 During online learning, I often ask myself questions to help me understand the learning content.

Q5 During online learning, I continually evaluate my learning outcomes to ensure I am progressing toward my goals.

Q6 During online learning, I frequently reflect on my learning methods.

Q7 During online learning, if I have developed a study plan, I follow it.

Q8 During online learning, I set both short-term and long-term learning goals.

Q9 During online learning, I break down a large problem into smaller tasks to complete it.

Q10 I can identify and improve the problems in my online learning process.

Q11 During online learning, I promptly adjust my study content and progress according to my learning situation.

Q12 After online learning, I assess whether I have completed my learning tasks and achieved my learning goals.

Q13 When my online learning outcomes are not ideal, I actively analyze the reasons and make adjustments.

**Online Learning Resource Management Strategies (OLRM)：**

Q1 During online learning, I can choose the study time that suits me best.

Q2 During online learning, when encountering difficulties, I seek help from peers.

Q3 Even if I find the learning resources provided by the online learning platform dull, I persist in completing my studies.

Q4 During online learning, despite not liking the content of expert lectures, I strive to learn for good grades.

Q5 I usually choose a place where I can focus to participate in online learning.

Q6 During online learning, when I find knowledge difficult to understand, I seek help from my tutor.

Q7 When encountering difficulties in online learning, I know which peers to seek help from.
